# How to drug a leukemic stem cell: deciphering heterogeneity for better specificity^∗^

**DOI:** 10.1016/j.bneo.2025.100146

**Published:** 2025-07-25

**Authors:** Alice Worker, Nicholas Jinks, Peter Woodmancy, Claire Seedhouse, Sophie G. Kellaway

**Affiliations:** Blood Cancer and Stem Cells, Centre for Cancer Sciences, School of Medicine, University of Nottingham, Nottingham, United Kingdom

## Abstract

Blood cancers, such as acute myeloid leukemia (AML), are becoming increasingly common due to an aging population but remain challenging to treat. Relapse is the most important singular cause of treatment failure in AML, and up to half of patients relapse after chemotherapy or bone marrow transplantation. Relapse in AML is primarily due to a population of quiescent leukemic stem cells (LSCs) that shelter in the bone marrow. Chemotherapy hits actively proliferating AML blasts, but LSCs escape and can later re-enter the cell cycle to regenerate the leukemia. LSCs resemble hematopoietic stem cells, but variable and unique differences may allow for LSC-specific treatment. In this review, we summarize the unique biology of LSCs, considering both global and subtype-specific traits. We describe how heterogeneity, both between different AML subtypes and within the LSC compartment, has impaired efforts to find drug targets so far and how this is being resolved with technological advances such as single-cell sequencing. We elucidate which aspects of LSC biology determine possibilities for targeted treatment and the progress so far made toward therapies to prevent or treat relapse.

## Visual Abstract

**Figure:**
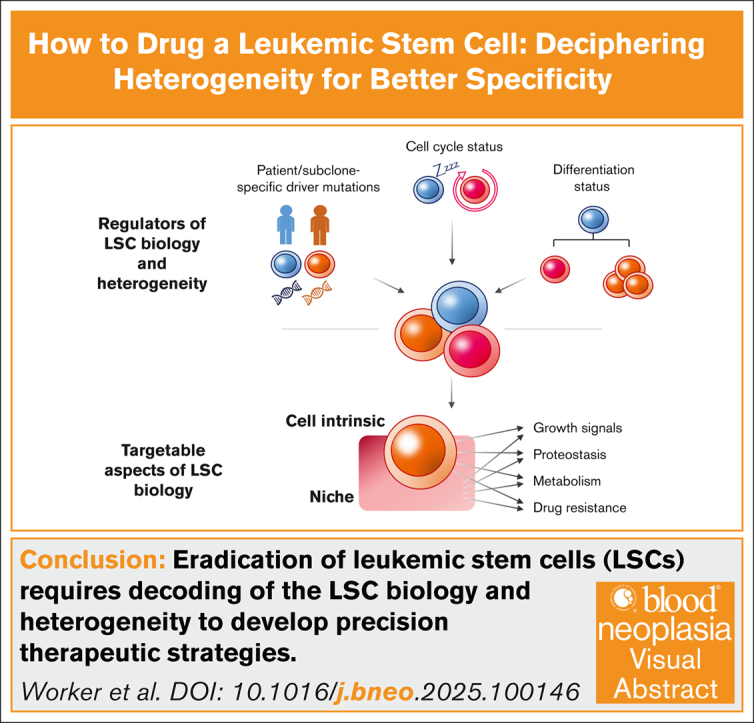

## AML is a heterogeneous blood cancer

Acute myeloid leukemia (AML) is a heterogeneous blood cancer characterized by the overproduction of immature myeloid blasts. Over the past 50 years, the standard treatment of newly acquired AML has been a regimen of intensive chemotherapy using cytarabine and anthracyclines. Complete remission is achieved in 60% to 85% of patients younger than 60 years and 40% to 60% of those older than 60 years. However, more than half of patients who undergo this intensive chemotherapy relapse within 1 year.^1^ Relapse is initiated by a pool of leukemic stem cells (LSCs) that are intrinsically resistant (primary resistance) or have developed resistance mechanisms in response to intensive chemotherapy (secondary/acquired resistance).

AML is driven by combinations of mutations in dozens of genes. Genes commonly mutated encode transcription factors (eg, core-binding factors), chromatin regulators (eg, DNA methyltransferase 3A [*DNMT3A*], Nucleophosmin [*NPM1*], and Lysine methyltransferase 2A [*KMT2A*] [formerly called MLL]), RNA-splicing regulators, and genes associated with intracellular signaling and metabolism (eg, FMS-like tyrosine kinase 3 [*FLT3*] and Isocitrate Dehydrogenase [*IDH1/2*]).^2^ The result of this complex mutational landscape is a highly heterogeneous disease, with certain mutations known to be associated with worse prognosis. AML mutational subtypes can be categorized as favorable, intermediate, or adverse prognosis depending on response to induction therapy.^1^ This heterogeneity, therefore, necessitates targeted approaches and in-depth analysis accounting for genotype. The success of FLT3 and IDH targeted inhibitors^3^ demonstrates the value of considering the driver mutations in AML, but other AML drivers have not been as amenable to targeting. Instead, 1 strategy to find novel drug targets is through detailed analysis of the transcriptional network resulting from the driver mutation and underpinning the disease.^4^^,^^5^ However, this precision medicine approach is further complicated by additional layers of heterogeneity both between individual patients and within the patient-specific cell population. Intrapatient heterogeneity can result from the presence of subclones,^6^ the stage of differentiation block^7^ and is also an integral aspect of the maintenance of disease, namely the leukemic hierarchy.

AML mirrors the hematopoietic hierarchy, in which primitive, self-renewing and generally quiescent cells sit at the apex and give rise to proliferative progenitors, followed by mature, specialized blood cells. However, in AML the dormant self-renewing cells—the LSCs—are those capable of initiating the leukemia, and of reinitiating the leukemia after chemotherapy, triggering relapse.^8^ High LSC frequency correlates strongly with reduced overall survival, whether patients receive chemotherapy or allogeneic stem cell transplant.^9^, ^10^, ^11^ The size of LSC population at diagnosis correlates with the percentage of drug-resistant cells after chemotherapy.^12^ Developing more effective treatment strategies, therefore, requires integrated analysis, to understand both the biology of the LSCs and the downstream impact of the driver mutations. Here, we review the current knowledge of targetable aspects of LSC biology and to what extent they are universal or subtype specific.

## Defining the LSC

Considerable efforts have been made to precisely determine what makes an LSC, and the optimal way to identify LSCs in patients. For developing true targeted therapies, there needs to be a clear and specific identification of the cells to be targeted. LSCs must be discriminated from both the highly proliferative AML blasts which are chemosensitive and any healthy hematopoietic stem cells (HSCs) present, which they otherwise resemble.

LSCs were originally identified by the cell surface markers CD34^+^/CD38^–^,^13^ with only this population of cells able to engraft in SCID mice. Some leukemic cells, a population typically carrying *NPM1* mutation, do not express CD34. In these *NPM1*-mutated leukemias, the LSCs can also be found in the CD34^–^ population.^14^^,^^15^ Furthermore, in later experiments, a minority of CD38^+^ cells have also been shown to engraft.^16^^,^^17^ These markers are therefore insufficient for isolating a complete and pure LSC population in all patients. To this end, several other surface markers have been investigated for their utility in purifying LSCs. CD90/Thy1 negativity is often used to enrich LSCs, particularly to distinguish from healthy HSCs^18^ with more AML-driver mutations found in LSCs sorted from 3 patients at adverse risk and 1 at intermediate risk by CD34^+^CD38^–^CD90^–^ as compared to the CD34^+^CD38^–^CD90^+^ sorted cells.^11^ However, CD90 expression is heterogeneous with evidence that CD90 positivity in blasts is associated with high risk/complex karyotype leukemias with poor outcomes.^19^ Other cell surface markers proposed for identifying LSCs have included CD45RA,^20^ TIM3,^21^ CD96^22^ and CD47,^23^ alongside lineage cocktail negativity. Purification of LSCs by cell surface markers is further complicated by the presence of LSC subtypes, resulting from their differentiation stage and cell cycle status.^24^^,^^25^ Due to the heterogeneity in driver mutations, cell stage and other individual patient-specific factors, no markers have yet been shown to conclusively identify a pure LSC population across all AMLs.

The differing markers utilized to identify LSCs has also created difficulty in the field of identifying secondary AML LSCs. Secondary AML can refer to leukemia that develops secondary to prior myelodysplastic syndrome, myeloproliferative neoplasm, or aplastic anemia, or as therapy-related AML.^26^ Within the current scope of the literature, secondary AML LSCs are not well-characterized or defined. Cells inferred to be otherwise healthy CD34^+^CD38^–^ stem/progenitor cells lacking the leukemic phenotype but with potential for leukemia initiation, termed “pre-LSCs” have been identified in patients with de novo AML. These pre-LSCs possess the AML-specific mutations found in blasts from the same patients such as *NPM1*, *TET2*, and *FLT3*-internal tandem duplication (*FLT3*-ITD).^27^ However, similar pre-LSCs have not yet been identified in these precursor syndromes. Nonetheless, a 2020 study did indicate that patients with secondary AML present with a higher fraction of CD34^+^CD38^–^ cells compared to patients with de novo AML,^28^ however further studies will ultimately be needed to determine whether these markers accurately identify true LSCs in patients with secondary AML who have progressed, or can identify pre-LSCs.

Identification of stem cells in healthy tissues or other cancers often makes use of the property of dormancy, with tracking via label retention. This technique has also been used with success in AML,^29^ but is typically less common. Challenges with defining LSCs by label retention include variable cell cycle status resulting from the significant plasticity of LSCs in response to their environment.^24^^,^^30^^,^^31^ Similarly, low reaction oxygen species (ROS) levels have been used to prospectively enrich LSCs linked to the proliferative and metabolic status of these cells,^32^ though ROS levels overall show high levels of heterogeneity.^32^^,^^33^ How variability in ROS levels relates to cell cycle status has not been explored. The reference standard for identifying LSCs is therefore, their capacity for leukemia initiation. Preferentially, this would be by identifying cells before relapse, in a residual disease state after treatment and after relapse.^34^^,^^35^ More typically, leukemia initiation is measured by engraftment in mice. However, engraftment in mice also has challenges, as interpatient heterogeneity in engraftment capacity is common and favorable-risk AML shows limited engraftment, which may or may not be directly associated with function of the LSCs.^36^, ^37^, ^38^

In vitro functional assays may also provide a clinically relevant method of evaluating LSC populations. Colony-forming assays using leukemic cells can detect patient-specific mutations and have shown significant prognostic value in predicting overall and event-free survival.^39^ These assays may therefore offer a high-throughput approach to evaluate stemness in leukemic cells that complements current LSC detection methods.

Given the challenges in isolating LSCs, recent advances in single-cell sequencing have proved valuable for studying LSCs without the need for perfect purification of LSCs by surface markers. Computational methods allow inference of a differentiation hierarchy,^40^^,^^41^ and separation of healthy and leukemic cells by tracking mutational burden.^42^^,^^43^ Moreover, single-cell methods have revealed significant heterogeneity within LSC populations, revealing different states of maturity and cell cycle/quiescence, both related to and independent of driver mutations.^41^^,^^44^, ^45^, ^46^, ^47^

## LSCs contribute to AML prognosis

**Figure 1.**
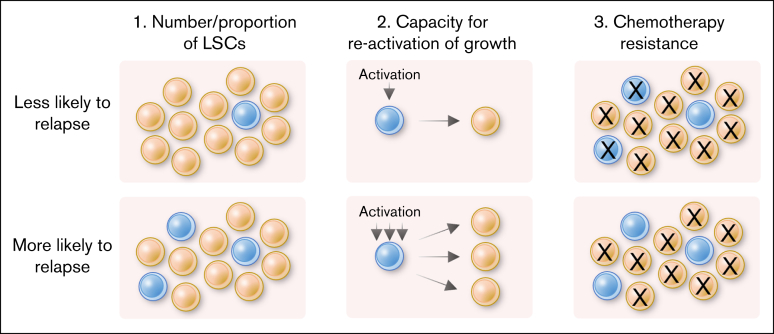
**Summary of potential factors underlying relapse likelihood.**

Considered together, the variability between LSC markers, prognosis and leukemia subtype raises the question of what causes some subtypes of AML to be more prone to relapse. We envisage 3 primary hypotheses as to how LSC features could be linked to disease prognosis and likelihood of relapse (Figure 1).

Firstly, if the proportion or total number of LSCs is greater, a patient is more likely to relapse. The overall number of LSCs can be associated with factors including patient health and immune function, whereas the proportion of AML cells which are LSCs is more likely to be related to disease-intrinsic factors. Patients with a high LSC burden at diagnosis have higher incidence of *TP53* and *FLT3*-ITD mutations, associated with intermediate/adverse prognosis.^11^ Similarly, the frequency of long-term leukemia-initiating cells classified functionally by in vivo engraftment was 5 to 7 times higher using cells from intermediate-/adverse-risk backgrounds compared to favorable.^48^ However, as described previously, variability in engraftment may result from differences in the characteristics of the cells or frequency of LSCs.

Secondly, prognosis may be associated with chemoresistance, which could influence both treatment failure and relapse. Several factors influence this, including drug resistance mechanisms, interaction of cells with the niche, the tumor microenvironment and the ability to enter a quiescent state. Thirdly, relapse could be influenced by how LSC growth is reactivated following chemotherapy, either by an external stimulus from the niche or surrounding cells, or by removal of the factors enforcing quiescence. Notably, all 3 scenarios are influenced by both the intrinsic biology of the leukemia and wider environment in which the leukemic cells sit. For example, single-cell RNA sequencing has shown that *DNMT3A* mutation alone, even in the clonal hematopoiesis setting, leads to upregulation of an LSC-associated gene signature in nonleukemic stem/progenitor cells.^49^ The intrinsic facets of LSC biology are discussed in detail in subsequent sections.

Given the complexity of these regulatory mechanisms, the degree to which driver-mutation–associated prognosis in AML is linked to total LSC burden or LSC features resulting from the driver-regulated transcriptional program is challenging to elucidate. This has led to the development of gene signature–based metrics associated with the clinical prognosis independent of other factors.^50^^,^^51^ The most widely used of these gene signatures is now the LSC17 score,^16^ a 17 gene stemness score. LSC17 score associates strongly with disease outcome and is generally used to indicate the proportion of LSCs present. Notably, LSC17 was not prognostic for some favorable-risk AML subtypes and led to the use of a RUNX1-based prognostic signature for core-binding factor AML.^52^ Similarly, the methodology has been refined for pediatric cohorts (LSC47 and pLSC6).^52^, ^53^, ^54^ This implies that the genes involved in determining an LSC signature are in some ways influenced by the underlying biology of the disease.

The LSC17 score measures gene expression and so may be influenced by the driver oncoproteins perturbing regulation of some, or all of the genes in the signature. Most genes in the LSC17 signature have not yet been functionally linked to LSC cell biology but several are preferentially expressed in certain AML subtypes (Table 1). Whether subtype-specific expression is due to differences in the functional characteristics LSCs or results from altered regulation by the driver is unclear. In 1 study, LSC17 score was higher in adverse-risk patients with t(6;9) AML than in favorable-risk *NPM1*-mutated AML, with the caveat that only 2 patients were studied from each subtype,^55^ suggesting a potential association with LSC burden or functional characteristics, as measured by LSC17, resulting from the AML driver. Similarly, using primitive cells enriched at relapse from pediatric AML, longitudinal single-cell multiomics (single-cell RNA and single-cell assay for transposase-accessible chromatin [ATAC] sequencing) highlighted driver-specific LSC transcriptional networks.^56^ This further exemplifies the need to consider the function of the driver in analysis of the LSCs. Comparisons of LSCs, at the single-cell level from differing AML backgrounds, overall reveal differences in gene signatures associated with relapse and predicted drug response.^44^^,^^57^^,^^58^

**Table 1.** **The genes comprising the LSC17 score and currently known functional associations**

Gene	Function in LSCs	Subtype association	References
*DNMT3B*	Methylation of stem cell–associated genes	Experiments performed in KMT2A::MLLT3, Myc-Bcl2 (mice)	^59^
*ZBTB46∗*	No evidence of functional role associated with LSCs	No evidence of subtype association	
*NYNRIN*	Mitochondrial regulation in HSCs, no evidence of functional role associated with LSCs	No evidence of subtype association	^60^
*ARHGAP22∗*	No evidence of functional role associated with LSCs	No evidence of subtype association	
*LAPTM4B*	No evidence of functional role associated with LSCs	FLT3-ITD/NPM1	^61^
*MMRN1*	No evidence of functional role associated with LSCs	No evidence of subtype association	
*DPYSL3*	No evidence of functional role associated with LSCs	No evidence of subtype association	
*KIAA0125*	No evidence of functional role associated with LSCs	RUNX1, inversely correlated with t(8;21) and t(15;17)	^62^
*CDK6∗*	Drives growth and activation of HSCs and LSCs	t(8;21), CEBPA, inversely correlated with inv(16); experiments in BCR::ABL1 (mice)	^63^,^64^
*CPXM1∗*	No evidence of functional role associated with LSCs	No evidence of subtype association	
*SOCS2*	Associated with LSC number and growth/quiescence control	Experiments performed in KMT2A::MLLT3, FLT3-ITD/NPM1c	^65^,^66^
*SMIM24∗*	No evidence of functional role associated with LSCs	No evidence of subtype association	
*EMP1*	No evidence of functional role associated with LSCs	Inv(16)	^67^
*NGFRAP1*	No evidence of functional role associated with LSCs	No evidence of subtype association	
*CD34*	Widely used cell surface marker, limited evidence of functional role in HSCs, no evidence of functional role associated with LSCs	Inconsistent expression with FLT3-ITD/NPM1	4,^68^
*AKR1C3∗*	No evidence of functional role associated with LSCs	No evidence of subtype association	
*GPR56*	Associated with intracellular signaling, affects engraftment in mice	EVI1-high; experiments carried out in HOX/MEIS (mice), FLT3-ITD/NPM1c	^69^,^70^

∗Genes with a negative influence on the score.

## Control of LSC growth and quiescence

Understanding of the unique biology of LSCs, with consideration for differences between both HSCs and blasts, is required to determine the association with prognosis and enable targeted therapies. Hematopoietic stem and progenitor cells would ideally be avoided to minimize side effects. Although drugs may also hit blasts without negative consequences, understanding the differences in how LSCs and blasts are regulated should reveal novel therapeutic targets. The most notable difference between LSCs and blasts is growth rate. Dormancy allows the LSCs to be protected from many chemotherapy agents which are only targeting rapidly proliferating cells. The LSCs present at both diagnosis and relapse show dormancy and a stemness gene signature,^34^ however, chemotherapy can also transiently cause LSC recruitment to cell cycle and induce alternative stem-like populations.^30^

**Figure 2.**
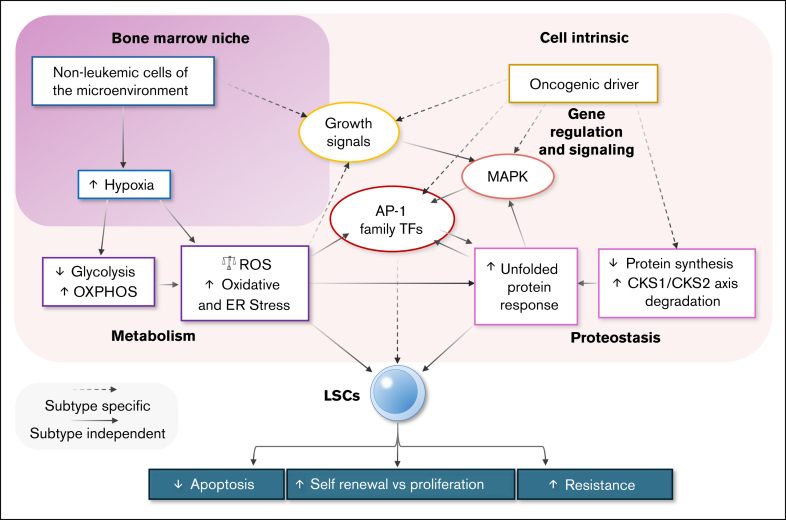
**Metabolism, proteostasis, and signaling together regulate the balance of growth, quiescence, and cell survival in LSCs.** Both cell-intrinsic (light pink background) and -extrinsic bone marrow niche factors (dark pink background) are able to influence a range of effects on LSCs, contributing to antiapoptotic and drug resistance promoting signaling. Dashed lines indicate mechanisms known to have subtype-specific regulation; solid lines indicate mechanisms for which no subtype-specific regulation known at this time. ER, endoplasmic reticulum; OXPHOS, oxidative phosphorylation.

Several factors control the balance between LSC quiescence and growth which may provide targetable pathways. LSC growth is influenced by regulation of growth factor signaling, metabolism, and proteostasis. Regulation of these processes can be global or subtype specific. These pathways show interconnectivity and joint dependence on key regulatory processes as shown in Figure 2, which we expand on throughout the remainder of this review. Specifically, protein turnover rate intrinsically regulated by the proteostasis axis influences the availability of amino acids for metabolism via oxidative phosphorylation, whereas oxidative phosphorylation itself increases ROS, which further impacts on the unfolded protein response. Moreover, growth factor signaling and the unfolded protein response feed into MAPK/Activator protein 1 (AP-1) signaling to regulate growth rate, which in turn further affects on the metabolic rate.

## Growth factor signaling

Hematopoiesis is intricately regulated by growth factor and cytokine signaling upstream of the MAPK and STAT pathways, and so it is perhaps unsurprising that AML co-opts these processes. Regulation of LSC growth is variable, with some facets global and others highly subtype specific. The *FLT3*-ITD mutation causes constitutive activation of the FLT3 tyrosine kinase, yet LSCs maintain quiescence despite this via mitochondrial accumulation and increased oxidative phosphorylation dependent on autophagy.^71^ Inhibition of autophagy could be a specific vulnerability in these cells but efficacy was reduced when combined with FLT3-targeted inhibitors suggesting the process could be specific to *FLT3*-ITD AML or associated with FLT3 signaling.^71^

Increased expression of the interleukin-3 (IL-3) receptor subunit *IL3RA* is commonly observed in LSCs,^72^ with aberrant ratios of specific IL-3 receptor subunits confirmed in single-cell RNA sequencing in quiescent LSCs of patients with *FLT3*-ITD and *RUNX1* mutations. This results in biasing of intracellular signaling in LSCs after binding to the receptor by IL-3 from the microenvironment,^73^ with the functional impact of IL-3 on leukemia initiation confirmed with serial colony formation. Similarly, upregulation of the IL-5 receptor subunit *IL5RA* was found specifically expressed in *GATA2*^+^ LSCs of= patients with t(8;21) AML using single-cell RNA sequencing, which along with vascular endothelial growth factor A (VEGFA)/VEGF receptor 2 converges on MAPK/AP-1 signaling.^46^ Supporting this, a gene signature including *GATA2* and *IL5RA* is associated with adverse prognosis and a significantly greater chance of relapse in t(8;21) patients.^74^ Both the IL-5 and vascular endothelial growth factor pathways could be targeted in LSCs, reducing engraftment in mice, with humanized monoclonal antibodies and suggesting feasibility of therapeutic blockade of LSC growth.^46^ Likewise, *PLCG1* is also upregulated in t(8;21) AML, and Ca^++^ signaling via AP-1 is essential for LSC survival and maintenance of self-renewal; targeting Ca^++^ signaling via cyclosporin A showed efficacy in mouse models.^75^ Interestingly, enforced overexpression of individual AP-1 transcription factors enriches for either label retaining cells or proliferative cells dependent on context, highlighting the complex but necessary interplay of signaling and the wider transcription network in modulation of growth.^33^

Secondary AML development following myeloproliferative neoplasms driven by aberrant JAK/STAT signaling—typically resulting from the JAK2 V617F mutation—has been recently characterized as displaying decreased interferon signaling and increased LSC-specific transcriptome signatures.^76^ This is thought to be due to repression of repetitive elements in the genome of LSCs, which downregulates interferon signaling and therefore, increases resistance against apoptosis and suggests a role for hypomethylating agents in blocking LSC survival.^77^

Subtype-specific growth factor signals derived from both the bone marrow microenvironment and LSCs themselves therefore activate intracellular signaling pathways, such as STAT/MAPK/AP-1, to promote LSC growth and resistance against apoptosis (Figure 2).

## Proteostasis

Protein homeostasis, or proteostasis, has been considered as a potential therapeutic avenue in AML due to HSC and LSC reliance on the process for maintained quiescence and long-term survival.^78^ Proteostasis involves a careful balance between protein synthesis, folding, trafficking, modification, and degradation. Long-term quiescent HSCs are characterized by a low rate of protein synthesis, which is controlled by posttranslational modification of factors such as 4E-BP1.^79^ Once HSCs begin to proliferate and differentiate, their protein synthesis rate increases.^78^ In parallel, LSCs present with highly regulated protein synthesis compared to blasts, with reduced expression of genes encoding translation machinery observed in t(8;21) AML LSCs,^46^^,^^80^ yet increased ribosomal biogenesis compared to mature cells.^81^ The specific regulatory mechanisms may underpin resistance against general proteasome inhibitors such as bortezomib and pevonedistat.^82^, ^83^, ^84^

LSC proteostasis is largely regulated by 2 signaling axes: the MAPK/AP-1 signaling axis and the Cyclin-dependent kinases regulatory subunit 1/2 (CKS1/CKS2) axis. The MAPK/AP-1 signaling axis enhances the expression of DUSP1 to promote the unfolded protein response in LSCs.^85^ The unfolded protein response is activated by genotoxic and endoplasmic reticulum stress induced by ROS and hypoxia, and is known to regulate healthy HSC self-renewal and high proteome quality.^86^, ^87^, ^88^, ^89^ Genes such as the MAPK signaling–responsive *JUN* (AP-1), heat shock transcription factor 1, and those in the HSP family, which are also overexpressed and promote survival of LSCs, are known to bind to unfolded protein response effector promoters. This results in high basal levels of unfolded protein response signaling, increasing LSC resistance to stress and apoptosis (Figure 2).^90^, ^91^, ^92^ The CKS1/CKS2 axis regulates HSC protein phosphorylation and ubiquitin-mediated degradation to sustain long-term HSC function^93^^,^^94^; low expression of CKS1 maintains HSC quiescence by limiting proliferation. LSCs from adverse-risk AML overexpress CKS1 compared to bulk AML cells and healthy HSCs, highlighting a specific vulnerability. CKS1 inhibition triggers LSC apoptosis, whereas healthy HSCs are protected by being pushed into quiescence.^95^ Disruption of the proteostasis axes on which LSCs depend to maintain quiescence, via targeted inhibition of unfolded protein response signaling, MAPK/AP-1 or CKS1, or the interconnected upstream and downstream pathways (Figure 2), could therefore be a promising therapeutic approach for targeting LSCs. Targeting these pathways may re-sensitize LSCs to proteostasis-related, hypoxia-influenced stress.

## Metabolic vulnerability

Specific metabolic pathways further control LSC proliferation and provide a route to therapeutic specificity. LSCs depend on oxidative phosphorylation for their energy and targeting mitochondrial oxidative phosphorylation has emerged as a promising approach for eliminating LSCs.

HSCs depend heavily on the hypoxic microenvironment of the bone marrow for their maintenance (Figure 2). HSCs rely on both anaerobic glycolysis and oxidative phosphorylation metabolism to meet their energy needs, the former primarily during quiescence and the latter during differentiation and proliferation.^96^^,^^97^ When quiescent, glycolysis allows low production of ROS, maintaining cellular self-renewal and minimizing ROS-associated oxidative damage.^98^ Conversely, oxidative phosphorylation typically generates more ROS than glycolysis, risking increased oxidative stress. However, LSCs typically depend on metabolism of amino acids for oxidative phosphorylation, facilitating greater proliferative potential and invasive properties,^32^^,^^99^^,^^100^ yet harbor low levels of ROS^32^ and may in some situations, be protected from oxidative stress.^101^^,^^102^ The reliance on, and activation of, oxidative phosphorylation in LSCs can contribute to self-renewal^100^ and can potentiate oncogenic signaling from driver mutations such as *FLT3*-ITD.^103^ Reduction in glycolysis may limit the effectiveness of some chemotherapeutic agents even in cells not otherwise showing hallmarks of LSCs.^104^ Single-cell RNA sequencing in a pediatric cohort enriched for core-binding factor AML showed enrichment of an oxidative phosphorylation signature after relapse in LSCs and transitional progenitors implying a role in driving leukemic regrowth.^105^

Direct modulation of ROS and oxidative phosphorylation through associated metabolic enzymes such as reduced NADP oxidases or electron transfer chain components may therefore, offer a novel therapeutic approach to exploit the pro-oxidative environment of LSCs.^99^^,^^106^, ^107^, ^108^ Given that the unfolded protein response is also influenced by ROS levels, therapeutic blockade of the oxidative phosphorylation on which LSCs rely for metabolism may also synergistically target proteostasis-related stress (Figure 2). Furthermore, the potential for high ROS environments as LSC growth is stimulated, mandate effective DNA repair systems raising the possibility of synthetic lethality.^102^

## Chemoresistance and novel targeting approaches

Beyond control of growth status, LSCs may also show drug resistance which must be factored into treatment development. The adenosine triphosphate binding cassette (ABC) transporter family of proteins regulate the export of many cytotoxic drugs from the cytoplasm to prevent DNA damage. Anthracycline export is regulated by several ABC transporters and their overexpression has been associated with chemoresistance in several types of cancer. High expression of ABCB1, ABCC1, ABCC3, and ABCB5 have been linked to multidrug resistance. LSCs exhibit increased expression of ABC transporters compared to more committed leukemic cell populations, which may contribute to their ability to persist after chemotherapy.^109^, ^110^, ^111^ Specifically, significantly increased expression of ABCB1 and ABCG2 in refractory patients is correlated with an increase in daunorubicin resistance and can be reversed by treatment with ABC transporter inhibitors.^110^^,^^112^

Targeting mitochondrial B-cell lymphoma 2 (Bcl-2) has shown promise as an LSC-directed therapy in AML and the Bcl-2 inhibitor venetoclax is the only LSC-directed therapy currently in clinical use.^113^, ^114^, ^115^ Multiple mechanisms of action have been proposed for how venetoclax targets LSCs downstream of Bcl-2 inhibition, including depletion of amino acids, suppression of oxidative phosphorylation and disruption of energy metabolism.^32^^,^^99^^,^^116^ The addition of venetoclax to treatment regimens has improved patient outcomes, particularly in older/unfit patients, however relapse is still common. LSC resistance has been attributed to reliance on other antiapoptotic family members,^117^ evasion of apoptosis by developing antiapoptotic mutations (*TP53*, myeloid cell leukemia 1 [*MCL1*], and *BAX*),^118^, ^119^, ^120^ or compensating through fatty acid metabolism.^99^ Notably, *RAS* mutations in LSCs specifically drive resistance to venetoclax, due to altered regulation of *BCL2* and associated genes^118^^,^^121^ with single-cell RNA sequencing highlighting that this was due to a shift toward a monocytic state induced by the RAS mutation. CRISPR/Cas9 screens on *FLT3*-ITD, *MLL*-fusion, or *NPM1*-mutated cell lines and xenografts have demonstrated that components of the mitochondrial translation machinery, proteins associated with the mitochondrial outer membrane, or the transcription factors regulating these can induce resistance to venetoclax.^122^, ^123^, ^124^, ^125^

Targeting resistance mechanisms with additional therapeutics is therefore high on the agenda. Synergistic agents to enhance the efficacy of venetoclax to eliminate LSCs are a current priority.^126^ For example, studies using different Mcl-1 inhibitors alongside venetoclax have shown synergistic action in primary LSCs, venetoclax-resistance cell lines, and xenograft mouse models.^127^, ^128^, ^129^ Furthermore, dual Bcl-2 and Mcl-1 inhibition has also been able to overcome resistance induced by *TP53* mutations,^130^^,^^131^ but the safety and efficacy of combination therapy with venetoclax has yet to be determined in patients.^132^ Recent data have shown that targeting the N6-adenosine-methyltransferase 70 kDa subunit (METTL3) with the small molecule inhibitor STM2457 enhances venetoclax sensitivity and can overcome venetoclax resistance by downregulating Mcl-1 and MYC protein stability.^133^ METTL3 inhibition reduces re-engraftment of MLL-AF6 LSCs in vivo^134^ suggesting that N6-methyladenosine RNA modifications catalyzed by the METTL3-14 complex are necessary for LSC maintenance.

Once again, however, drug targeting and resistance is complicated by the layers of heterogeneity present in LSCs based on cell cycle and differentiation state.^25^^,^^135^ Leveraging cellular indexing of transcriptomes and epitopes by sequencing (CITE-seq) Leppä et al^136^ were able to determine subclonal LSC therapeutic vulnerabilities, giving an insight into what the next generation of personalized LSC-targeting therapies could look like.

## Conclusions

Eradication of LSCs requires a careful precision medicine approach. Although clinical success has been seen with venetoclax, resistance and compensatory mechanisms require additional target development. Many aspects of LSC growth and survival depend on the regulatory network controlled by the driver oncogenes, with control of growth rate through oxidative phosphorylation and proteostasis showing nuanced regulation. None of the facets regulating LSC quiescence, self-renewal, growth, and survival occur in isolation. Instead, a high degree of crossregulation between growth factor signaling, metabolism, and proteostasis is seen (Figure 2). This may allow for joint targeting of several pathways controlling LSC survival and growth or could lead to compensatory mechanisms when targeting 1 facet of the biology alone. For example, synergistically targeting the unfolded protein response and oxidative stress converges to induce apoptosis in *FLT3*-ITD/*MLL*-fusion AML cell lines.^137^ This suggests that what is required is an approach targeting multiple factors, both extracellular and intracellular, which govern LSC survival. Significant interpatient and intrapatient heterogeneity exists with the LSC populations, both in terms of size and behavior. Identifying and decoding subpopulations of LSCs in both de novo and secondary AML, with respect to the wider transcriptional landscape, may be the next step toward a cure but requires consideration of all pathways on which LSCs rely, together.

Conflict-of-interest disclosure: The authors declare no competing financial interests.
